# Advances in Inorganic Nanomaterials for Triboelectric Nanogenerators

**DOI:** 10.1021/acsnanoscienceau.1c00026

**Published:** 2021-10-27

**Authors:** Renyun Zhang, Håkan Olin

**Affiliations:** Department of Natural Sciences, Mid Sweden University, SE85170 Sundsvall, Sweden

**Keywords:** Triboelectric nanogenerators, Nanomaterials, Inorganic nanomaterials

## Abstract

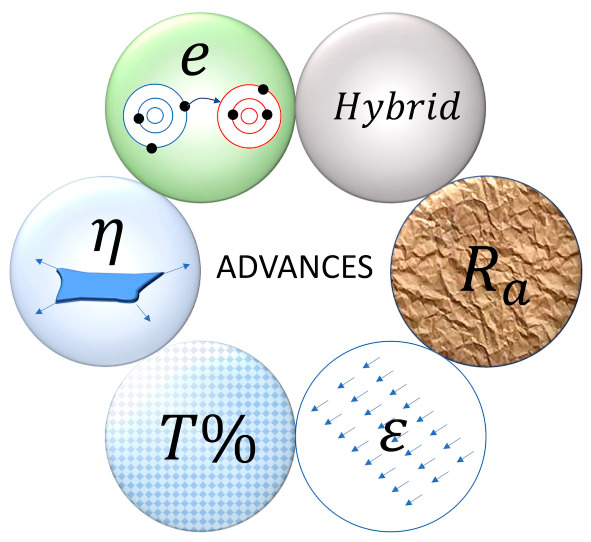

Triboelectric nanogenerators (TENGs) that utilize triboelectrification and electrostatic induction to convert mechanical energy to electricity have attracted increasing interest in the last 10 years. As a universal physical phenomenon, triboelectrification can occur between any two surfaces that experience physical contact and separation regardless of the type of material. For this reason, many materials, including both organic and inorganic materials, have been studied in TENGs with different purposes. Although organic polymers are mainly used as triboelectric materials in TENGs, the application of inorganic nanomaterials has also been intensively studied because of their unique dielectric, electric, piezoelectric, and optical properties, which can improve the performance of TENGs. A review of how inorganic nanomaterials are used in TENGs would help researchers gain an overview of the progress in this area. Here, we present a review to summarize how inorganic nanomaterials are utilized in TENGs based on the roles, types, and characteristics of the nanomaterials.

## 1 Introduction

The invention of triboelectric nanogenerators (TENGs) in 2012^1^ turned the historic physical phenomenon of triboelectrification (contact electrification)^2^ into a working principle for energy conversion. With the development of TENGs, additional applications^3^ to energy conversion have been discovered, including sensors,^4−8^ control interfaces,^9^ functional systems,^10^ and biomedical applications.^11,12^ The nature of triboelectrification implies that it can occur between any two materials that have physical contact and separation, as charges can transfer between the surfaces. For this reason, diverse materials have been studied for use in TENGs with different purposes, utilizing unique physical and chemical properties.

There are many review articles^13−24^ that have described the working modes, mechanisms, and applications of TENGs. Zheng et al. have reviewed the application of TENGs in biomedical applications.^13^ Chen and co-workers^14^ and Wang et al.^20^ have reviewed energy harvesting and self-powered sensing using TENGs. Wang^15,16^ has reviewed the theoretical progress of triboelectrification. Zhang and Olin^17^ and Bai et al.^19^ have reviewed the materials that are utilized in TENGs. Pan and co-workers^25^ have reviewed the applications of TENGs for future soft robots and machines. In this Review, we skip this part of the information and focus solely on how inorganic nanomaterials are used to improve the performance of TENGs.

The performance of TENGs is highly dependent not only on the triboelectric materials and their dielectric properties but also on how to pair two triboelectric materials. With the aim of boosting performance, nanomaterials have been introduced to TENGs. Nanomaterials can serve as either electrode or triboelectric materials, depending on the types of nanomaterials and how they are utilized. Nanomaterials in all dimensions have been applied to TENGs with significant performance improvements.

This Review summarizes the utilization of nanomaterials via several aspects: (1) the roles of inorganic nanomaterials, (2) the types of inorganic nanomaterials, and (3) the composition of the nanomaterials. Perspectives of future studies are also given after the summary. This Review provides an overview of how inorganic nanomaterials that are used in TENGs can lead to further development in this area.

## 2 The Roles of Inorganic Nanomaterials in TENGs

The roles of nanomaterials can be divided into two categories: electrode materials and triboelectric materials. Metallic nanomaterials and some carbon-based nanomaterials have been used as electrode materials in TENGs because of their excellent electrical properties.

**Figure 1:**
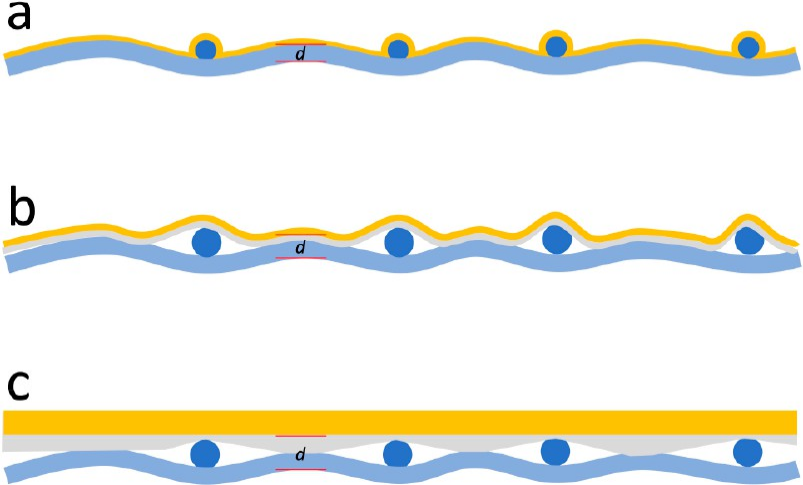
Different electrodes have been used in TENGs. (a) Thermally evaporated metallic nanofilms, (b) glue-based electrode attachment, and (c) metallic tape-based electrode attachment. The *d* in the figure shows the electrostatic induction distance of the TENGs.

The differences in physical and chemical properties of the triboelectric materials place great importance on electrode selection. The most common nanomaterials used as electrode materials are thermally evaporated gold^26^ or copper^27^ nanofilms at the backside of triboelectric materials. Such deposited nanofilms have a larger contact area (Figure 1) between the electrode and the triboelectric material, which can enhance electrostatic induction. These nanofilms are of critical importance when materials with high roughness such as textiles^28^ are used as triboelectric materials. Glue- or tape-based electrode attachment methods (Figure 1) limit the contact area between the electrode and the triboelectric surfaces. Moreover, the direct deposition of a nanofilm eliminates the glue layer, which reduces the distance of electrostatic induction, resulting in a higher current output for the TENGs.

In addition to metallic materials,^29^ carbon materials such as graphene^30^ have also been used as electrodes in TENGs due to their excellent electrical conductivity, flexibility, and optical properties. Different techniques have been used to produce graphene, including chemical vapor deposition (CVD),^27,30−33^ laser induction,^34,35^ layer-by-layer assembly,^36^ and doping.^37^ Carbon nanotubes^31,32,38−40^ have also been applied as electrodes in different forms. Carbon nanotubes could serve as pure electrodes in the form of network layers or be embedded in polymers^38−40^ to enhance the flexibility of the TENGs.

The utilization of inorganic nanomaterials to improve the performance of TENGs has gained the most interest from researchers. The reasons are as follows: (1) surfaces with inorganic nanomaterials have different charge distributions on flat surfaces; (2) the high surface energy of inorganic nanomaterials can enhance the charge transfer between triboelectric surfaces; (3) the dielectric properties of inorganic nanomaterials can change the properties of composites containing inorganic nanomaterials; and (4) nanomaterials can bring extra characteristics to TENGs that can promote their application in specific areas. Of course, there are other advantages that nanomaterials have brought to TENGs. We review the utilization of inorganic nanomaterials below.

## 3 Inorganic Nanomaterials Used in TENGs

Inorganic nanomaterials that have been studied in TENGs include metallic nanomaterials, metallic oxides, 2D materials, perovskites, ferroelectric nanomaterials, and carbon nanomaterials. The utilization of these nanomaterials is due to their electrical, dielectric, and surface topographical properties; e.g., metallic nanomaterials have good conductivity, metallic oxides such as TiO_2_ have strong positive charge affinities, 2D semiconductors have flat surfaces and mechanical flexibilities, and ferroelectric nanomaterials can provide piezoelectricity.

### 3.1 Metallic Nanomaterials

#### 3.1.1 Gold Nanomaterials

**Figure 2:**
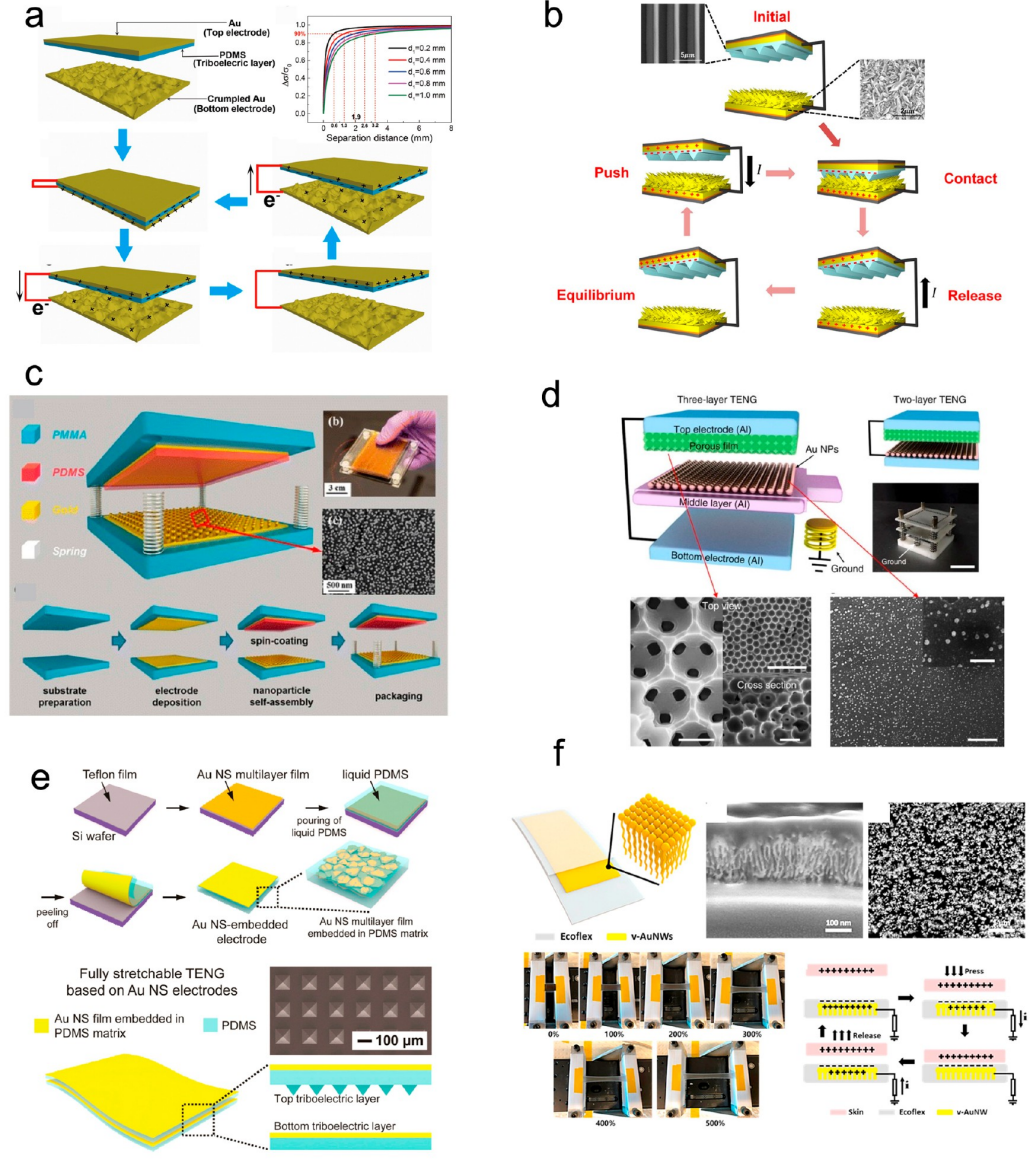
Gold nanomaterial constituted TENGs. (a) Crumpled gold film-based TENG. Reproduced from ref (41). Copyright 2018 Elsevier. (b) Gold nanoflower-based TENG. Reused under CC BY 4.0 (ref (42)). (c) Gold nanoparticle-based TENG. Reproduced from ref (44). Copyright 2013 American Chemical Society. (d) Gold nanoparticle-based TENG. Reused under CC BY 4.0 (ref (43)). (e) Embedded gold nanosheet-based TENG. Reproduced from ref (46). Copyright 2017 Elsevier. (f) Gold nanowire-based TENG. Reproduced from ref (48). Copyright 2020 Elsevier.

Gold nanofilms are commonly used metallic nanomaterials as electrodes in TENGs due to their excellent conductivity and low contact resistance. In addition to the direct thermal deposition methods reviewed above, a sputter coat of gold on a prestretched elastomer film could result in a crumpled gold electrode.^41^ Such a crumpled electrode (Figure 2a) could increase the contact area between the gold and the countertriboelectric layer. Such an advance has led to a maximum open-circuit voltage of 124.6 V, a maximum current of 10.13 μA, and a power density of 0.22 mW/cm^2^. The same increment of contact area has also been found on nanoflowers (Figure 2b), similar to gold electrodes deposited by electrochemical methods.^42^ The top performances of the TENG are 110 V, 5.5 μA, and 150 μW/cm^2^.

Different from the gold nanofilms reviewed in the above section, where the films are used only as electrodes, gold nanofilms have also been used as triboelectric layers.^26^ The reasons metallic films are used as both triboelectric layers and electrodes are their excellent electric conductivity, high permittivity, and charge affinity.^17^ The presence of gold nanoparticles could increase the contact area^43^ with the counterlayer, such as polymers, and enhance the stability of the output due to the high oxidation resistance of gold. The output power of a TENG (Figure 2c) in the presence of a 56 nm gold nanoparticle layer was enhanced 25 times^44^ due to the increase in contact area and nature of the positively charged gold nanoparticle surfaces. An area power density of 313 W/m^2^, a volume power density of 54 268 W/m^3^, and a maximum open-circuit voltage of 1200 V were obtained on the TENG.

In addition to the nanofilms, gold nanoparticles have also been impregnated in PDMS mesoporous pores^45^ and served as triboelectric layers to generate charges with a PDMS (Figure 2d). The impregnation of gold nanoparticles enhanced the power of TENGs over 5-fold compared to a flat film. Similar to this impregnation process, gold nanosheets have been embedded in a PDMS film^46^ to enhance the stability and stretchability of TENGs (Figure 2e) and in a PTFE film to enhance the surface charge density and the output current.^47^ The stretchability of TENGs brought by the gold nanosheet allows them to be applied at the joints of the human body for energy harvesting and sensing. Tactile sensors have been made with embedded gold nanostructures^48^ with potential application in human–machine interactions (Figure 2f).

Table 1 summarizes the performances of the gold nanomaterial constituted TENGs, showing the nanomaterials, countertribolayers, open-circuit voltages, short current or current densities, and power densities.

**Table 1:** Performances of gold Nanomaterial Constituted TENGs

gold nanostructures	counter tribolayer	open-circuit voltage (V)	current or current density	power density (mW/cm^2^)	ref
crumpled gold film	PDMS	124.6	6.75 μA/cm^2^	0.22	(41)
gold nanoflower	PDMS	110	1.53 μA	0.15	(42)
gold nanoparticles	PDMS	300	1.22 mA	46.8	(43)
gold nanoparticles	PDMS	∼1200	2 mA	313	(44)
gold/pdms	Al	150	0.62 μA/cm^2^	0.16	(45)
gold nanosheets	PDMS	60	2.8 μA		(46)
gold nanowire	skin	0.1–0.2			(48)

#### 3.1.2 Silver Nanomaterials

Silver is another widely used metallic material in TENGs. Compared to silver nanoparticles, silver nanowires are more commonly used in TENGs. Silver nanowires are popular because they can be simply transformed into transparent, flexible, and low-resistance electrodes for use in TENGs. Another reason is that the methods for the synthesis of silver nanowires are generally simple. Table 2 summarizes the performances of the silver nanomaterial constituted TENGs.

**Table 2:** Performances of Silver Nanomaterial Constituted TENGs

silver (Ag) nanostructures	counter tribolayer	open-circuit voltage (V)	current or current density	power density (mW/cm^2^)	ref
Ag nanoparticles	FEP	200	20 μA	0.11	(49)
Nano-Ag ink	Kapton	160	6.6 μA/cm^2^	1.2 mW/cm^3^	(50)
Ag NW	Al/Skin	66	8.6 μA	0.0446	(49)
Ag/PEDOT:PSS	PUA	170	50 μA	1.5	(50)
Ag NW	FEP	150	7.5 μA	0.036	(54)
Ag NW/PDMS	PFA	120^a^	22 μA^a^		(55)
	Nylon	18^a^	3.5 μA^a^		
Ag NW (as a transparent electrode)		3600	7 μA		(56)
Ag NW (as an electrode)		330	15.5 μA	0.25	(57)
Ag NW/PVDF	Nylon	240	12 μA^a^		(58)

a Data read from the figures in the paper.

Yang’s group reported a silver nanoparticle film-based TENG^49^ for harvesting energy from wind, where a silver nanoparticle film pasted on photograph paper was used as both an electrode and a triboelectric material. The positive charge affinity nature of the silver nanoparticles offers a rapid charging and discharge process and thus high output performance for a TENG. Brushing nanosilver ink^50^ on photopaper has also been used to create a silver film for use in TENGs.

Although the enhancement of silver nanoparticles has been proven, more studies have utilized silver nanowires since more features, as described above, could be created. Example features are the flexibility and transparency of silver nanowire-based electrodes.^51^ These features are of great importance for the application of TENGs as sensors to be attached to the human body.^52,53^

**Figure 3:**
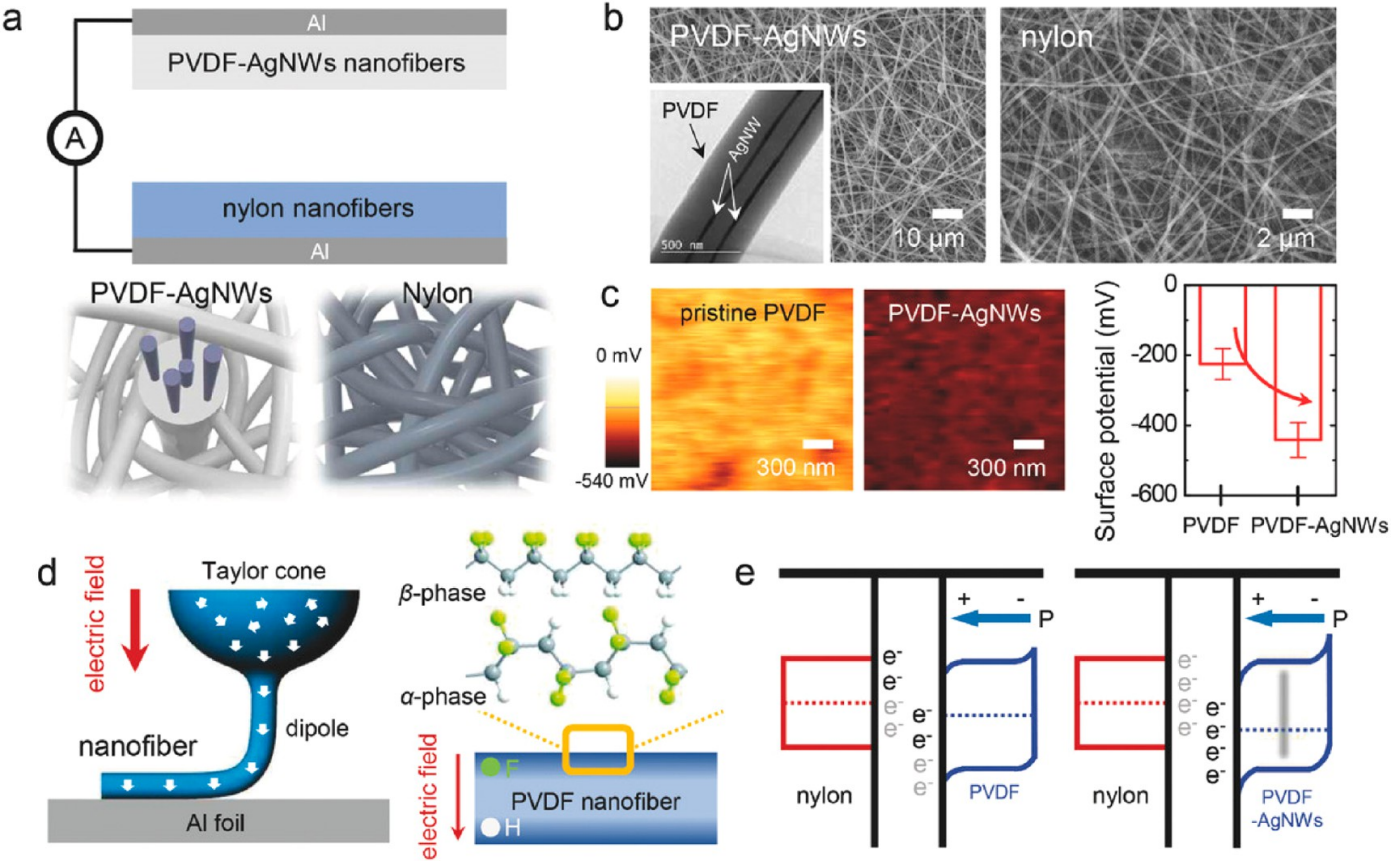
(a) Schematic diagrams of the TENGs based on the PVDF–AgNW composite and nylon nanofibers prepared by electrospinning methods. (b) SEM images of the electrospun PVDF–AgNW composite and nylon nanofibers. The inset shows a TEM image of the PVDF–AgNW composite nanofibers. (c) KPFM images of the surfaces of the pristine PVDF and PVDF–AgNW composite nanofibers. The right panel shows the surface potentials of the nanofibers. (d) Schematic illustration of the electrospinning process applied to a PVDF solution. (e) Schematic band diagrams explaining the TENG operation mechanism. Reproduced from ref (58). Copyright 2020 Wiley-VCH Verlag.

Pasting,^54^ spin coating,^52^ Meyer-rod coating,^55^ and splashing^56^ are commonly used methods for creating silver nanowire electrodes, as these methods allow easy control of the thickness of silver nanowire films. To improve the performance of the electrodes, an embedding technique^57^ has been used to fix the silver nanowire networks inside polymers such as PDMS,^46^ PVDF (Figure 3),^58^ and PEDOT:PSS^53^ so that the contacts among the silver nanowires will not be lost during physical deformation. The embedding of the silver nanowires maximizes the contact between the polymer, which enhances the electrostatic induction of the TENGs. To further increase the stability of the performance of the TENGs, researchers welded silver nanowires^52,59^ before embedding.

### 3.2 Metallic Oxides

Metallic oxides such as TiO_2_ and ZnO have high positive charge affinities, making them easy to pair with materials with negative charge affinities. The tunable surface morphologies and the work functions of these materials can ultimately affect the electrical performance^61^ of TENGs. In addition, the permittivity of the metallic oxides^62^ could also have an impact on the charge transfer process of the TENGs. Table 3 summarizes the performances of some of the TENGs constructed with metallic oxides.

**Table 3:** Performances of Metallic Oxide Constituted TENGs

metallic oxide	counter tribolayer	voltage (V)	current	power	ref
ZnO	PI	31.6	5.43 μA		(63)
ZnO (Ga-doped)	PDMS	27	119 nA/cm^2^		(64)
ZnO (Sb-doped)	Nylon	12	110 nA/cm^2^		(60)
ZnO	PTFE	28	4.5 μA	80 μW/cm^2^	(61)
ZnO	Ag	120	65 μA	1.1 mW^a^	(65)
ZnO/MWCNT/PDMS	Al	140.81	6.10 μA	0.26 W/cm^2^	(67)
ZnO	PI	80	0.9 μA^b^	1.12 W/m^2^	(68)
ZnO	PTFE	57	59 mA/m^2^	1.1 W/m^2^	(69)
ZnO/BC	Teflon	57.6	5.78 nA	42 mW/m^2^	(70)
ZnO/PVDF	PTFE	119	1.6 μA	10.6 μW/cm^2^	(71)
TiO_2-x_/PDMS	PDMS	180	8.15 μA	1.84 W/m^2^	(75)
TiO_2_/rubber	PTFE	113	9.8 μA	237 mW/m^2^	(76)
Fe_3_O_4_/PVDF	Al	138	5.68 μA		(82)

a Four layer stacked.

b Data read from the figure in the paper.

#### 3.2.1 ZnO Nanomaterials

The tunable surface morphologies of ZnO make it possible to design specific structures for TENGs. Nanoparticles,^63^ nanorods,^60,61,64−66^ nanoflowers,^67^ nanoripples,^68^ and microballon arrays^69^ have been created and used in different TENGs. The open-circuit voltage of TENGs using ZnO nanostructures is usually lower than 100 V,^61,63,64,70^ while the number can be improved to above 100 V^71^ to several hundred volts^72^ if specifically composited with polymers.

**Figure 4:**
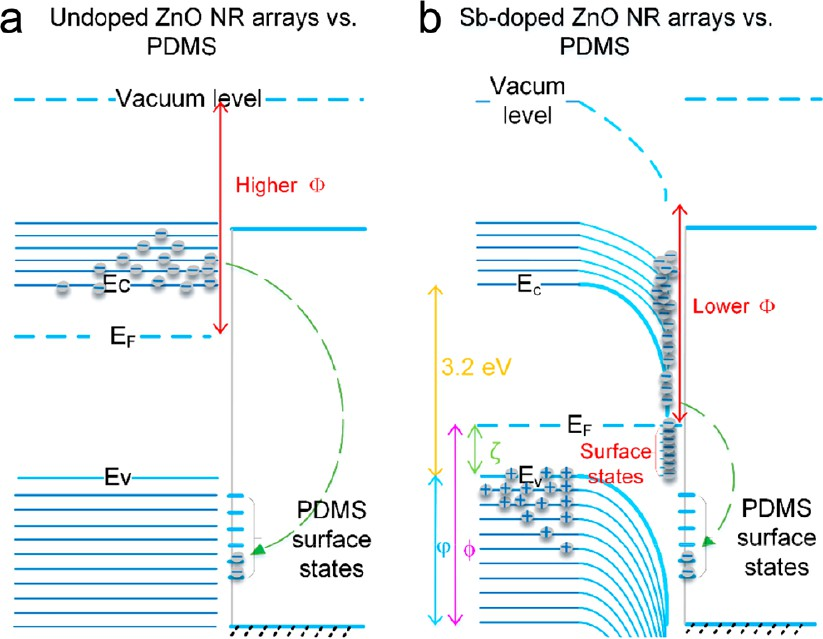
Proposed possible surface energy downward bending diagrams and tribocharge transfer for the TENG made of (a) undoped ZnO NR arrays and (b) Sb-doped ZnO NR arrays against PDMS before contact. Reproduced from ref (60). Copyright 2018 Elsevier.

One unique feature of using ZnO nanomaterials is that one can dope the materials to change their electrical behaviors. A TENG performance boosting effect can be brought by charge transfer at the surface due to doping.^60^ Chen et al. reported that 4 M % Sb-doped ZnO nanorod arrays can boost the output voltage and current by 24 and 5.5 times compared to undoped arrays. Sb doping bends the band of the ZnO nanorod arrays downward (Figure 4), leading to more electrons being transferred to the countertriboelectric surface. A similar phenomenon has also been found on Ga-doped ZnO nanorod arrays.^64^ Another TENG performance boosting effect brought by doping is the high trap density close to the conduction band edge, which results in shallow trapped electrons.^73^

#### 3.2.2 TiO_2_ Nanomaterials

Similar to ZnO, TiO_2_ nanomaterials have tunable shapes and sizes. TiO_2_ nanoparticles^74−77^ have been more widely studied in nanogenerators. In most studies, TiO_2_ nanomaterials are embedded^75^ or composited^74^ with polymers to improve the TENG performance. The chemical modification of TiO_2_ nanostructures^78^ is another way to enhance the output of TENGs.

The high dielectric constant of TiO_2_ nanomaterials is one of the factors that improves the output performance of TENGs. Moreover, the oxygen vacancies^79,75^ in TiO_2_ composites have also been suggested as a factor of performance enhancement.

#### 3.2.3 Other Metallic Oxides

In addition to ZnO and TiO_2_, other metallic oxide nanomaterials, such ITO,^80^ Al_2_O_3_,^81^ and Fe_3_O_4_,^82^ have also been studied for their applications in TENGs. Chun et al.^80^ reported a TENG based on an interlocked array of surface-functionalized ITO nanohelixes and demonstrated an over 340 times output enhancement compared to a flat ITO. Such enhancement is attributed to the nanoscale roughness, negligible degradation against external force and strain, and efficient charge generation and induction. Kim’s group reported a stoichiometric Al_2_O_3_-based TENG^81^ that reveals meaningful electric power generation under mechanical friction.

### 3.3 2D Nanomaterials

The application of 2D nanomaterials has gained increasing attention from researchers due to their unique geometry and electrical and dielectric properties. Based on the difference in electrical and dielectric properties, 2D nanomaterials have been used as electrodes or triboelectric layers in TENGs.^83^

#### 3.3.1 Graphene and Graphene Oxide

Graphene is a monolayer of carbon atoms that exhibits excellent electrical conductivity. Such a property makes graphene a good alternative to metallic electrodes. Moreover, graphene is an inert material that makes it chemically stable under extreme conditions. The transparency of graphene brings more features to TENGs. From a sustainable perspective, graphene is a green material that can benefit the development of environmentally friendly TENGs.

Shankaregowda et al.^84^ reported a flexible and transparent TENG based on a graphene layer grown by a CVD method, in which graphene was used as both an electrode and a triboelectric layer. Such a TENG has maximum outputs of 650 V, 12 μA, and 0.21 mW/cm^2^. By crumpling the graphene layer,^85^ the output power density can reach 0.25 mW/cm^2^. By aligning the graphene sheets,^86^ the output power density reached 4.8 mW/cm^2^. The alignment of the graphene sheets forms numerous microcapacitors and has a low dielectric loss that contributes to a high output power density.

**Figure 5:**
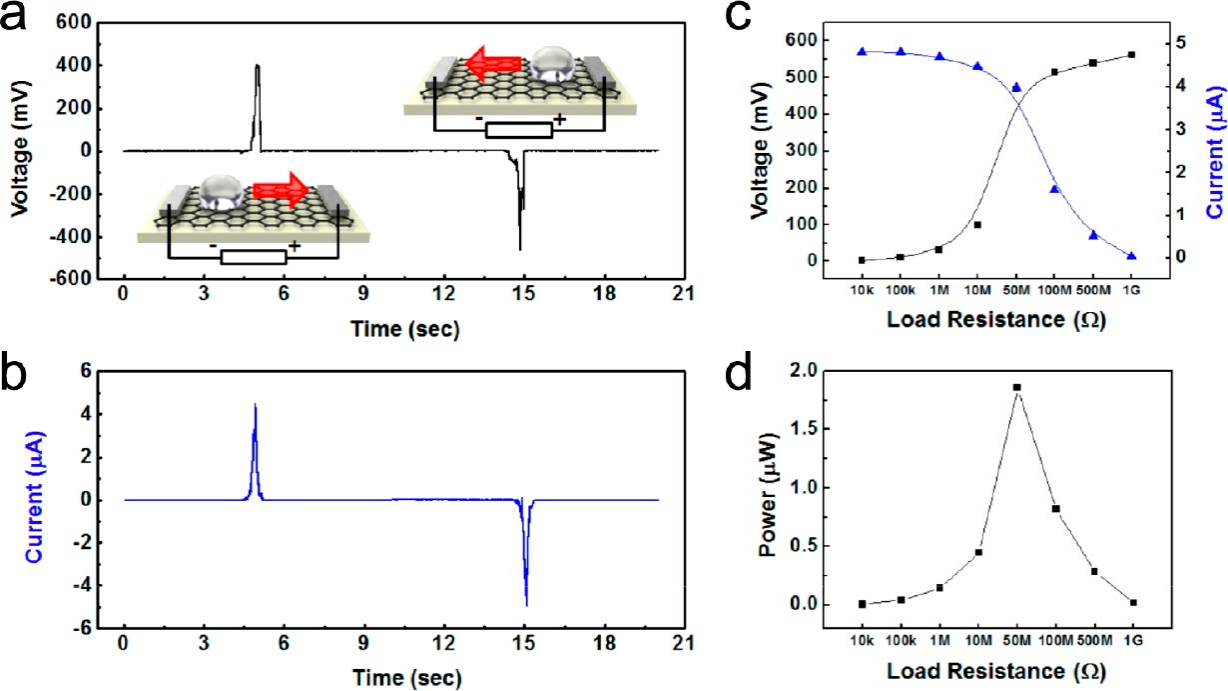
Water droplet-based electric power generation from a graphene/PTFE structure. (a) Voltage output from a graphene/PTFE structure by a single moving droplet of 0.6 M NaCl on a graphene surface with a reversed voltage signal output of a graphene/PTFE structure obtained by reversing the moving direction of the droplet. (b) Current output from the graphene/PTFE structure according to the forward and reverse motion of the water. (c, d) Dependence of the output voltage and current as a function of the external load resistance and corresponding maximum power output by a single moving droplet of 0.6 M NaCl, respectively. Reproduced from ref (87). Copyright 2016 American Chemical Society.

A graphene layer has also been applied to convert mechanical energy from water droplets. Kwak et al.^87^ reported a triboelectrification-induced large electric power generator based on a graphene/PTFE structure (Figure 5). The transfer of graphene on the PTFE surface polarized the graphene with negative charges accumulated on the top surface that interacted with the droplet to generate electricity. An output power of 1.9 μW from a single droplet was measured based on the unique graphene/PTFE structure.

Although CVD-grown graphene has excellent electrical conductivity, the production rate is still quite low due to the limitation of the technique. Alternatively, researchers have used reduced graphene oxide (rGO) in the development of TENGs. Usually, rGO is produced using thermal^88,89^ and optical^34^ methods. rGO can be used as either electrodes or triboelectric layers, depending on the design. Stanford et al.^34^ and Zhao et al.^35^ reported two separate studies on laser-induced graphene as electrodes. The first group used a 75 W pulse laser, while the second group used an 8.1 W continuous laser. Li et al.^90^ reported a TENG using thermally reduced rGO at 700 °C as a triboelectric layer and successfully converted the mechanical energy between the rGO and electrolyte into electricity. Zhao and co-workers^88^ reported a foam structure made from thermally reduced rGO and polyimide (PI) and used it as a pressure-sensitive electrode in a wind-driven TENG. Wu et al.^91^ reported the electron-trapping behavior of rGO embedded in a PI film, which can enhance the output approximately 30 times.

In some cases, GO has not been reduced for use in a TENG. Harnchana et al. reported a TENG made from a composite of GO, PDMS, and SDS^92^ that has an output of 438 V and 11 μA/cm^2^. Such performances are approximately three times those of a flat PDMS layer. GO can also be used directly as a triboelectric layer^93^ for energy harvesting and dynamic force sensing. Such a simple construction enabled the TENG to have a surprisingly high performance, with a short-circuit current density of 3.43 μA/cm^2^, an open-circuit voltage of 1100 V, and a power density of 3.13 W/m^2^. Moreover, the TENG can also act as a force sensor with a sensitivity of 388 μA/MPa.

#### 3.3.2 MXenes

MXenes are a class of 2D transition metal carbides, carbonitrides, and nitrides with compositions of M_*n*+1_AX_*n*_. MXenes are electronegative materials owing to their electric negative surface groups such as −OH and −F, making them candidate triboelectric materials for use in TENGs.^95^ Reports have indicated that MXenes can be more negative than PTFE.^96^

**Figure 6:**
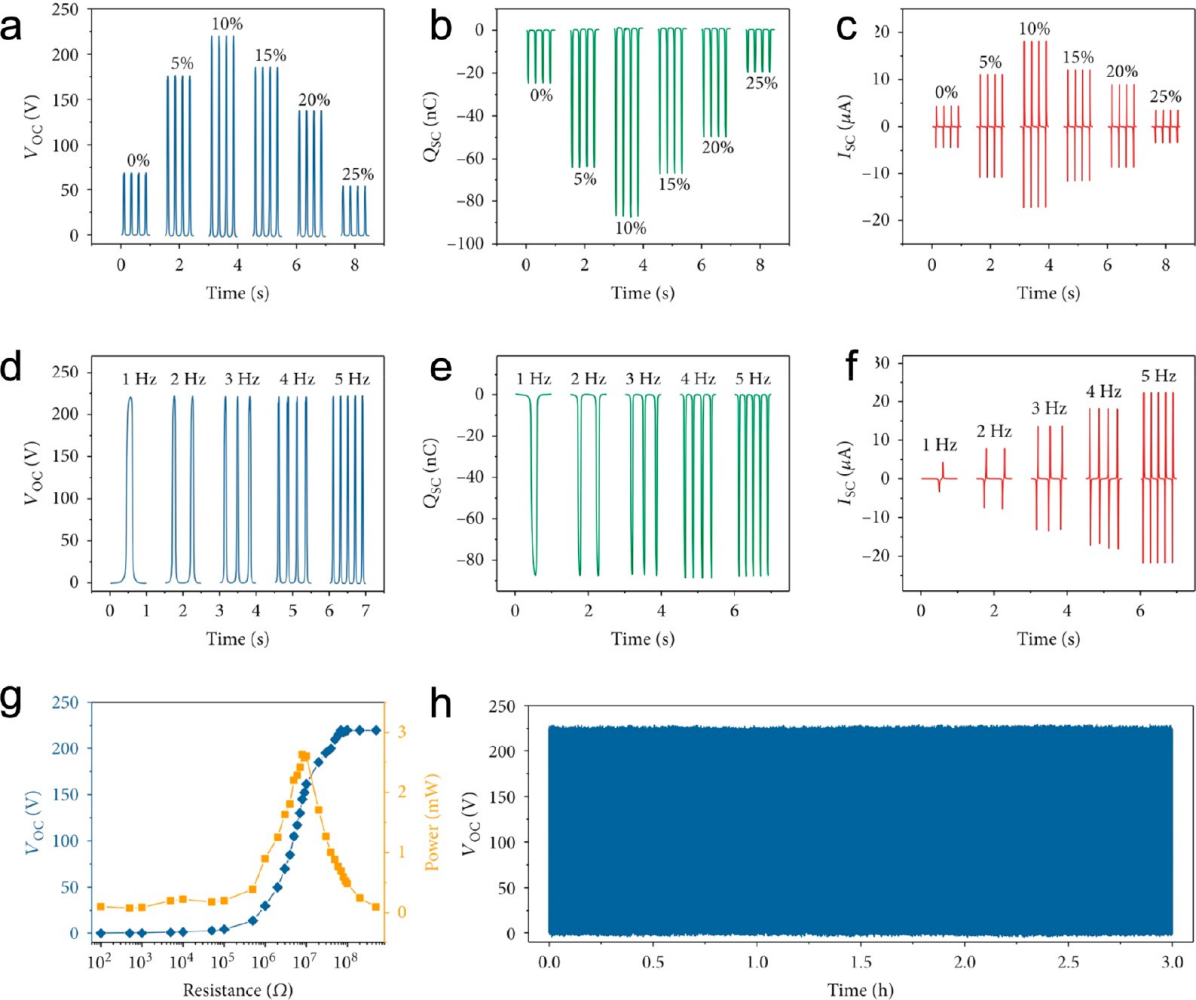
Triboelectrification of the MXene/PVDF membrane. (a–c) Open-circuit voltage (a), short-circuit transferred charges (b), and short-circuit current (c) of the TENG based on the MXene/PVDF membrane at different loadings ranging from 0% to 25%. (d–f) Open-circuit voltage (d), short-circuit transferred charges (e), and short-circuit current (f) of the TENG at an MXene loading of 10% operated at different frequencies (1–5 Hz). (g) Dependence of the output performance of the TENG on the external loadings. (h) Stability test of the fabricated TENG based on the MXene/PVDF membrane. Reference (94), reused under CC BY 4.0.

Due to the strong electronegativity of MXenes that attract electrons during triboelectrification processes, the open-circuit voltage of TENGs based on MXenes is usually high. For example, a TENG with a structure of MXene/glass:PEI-ITO^97^ can have an open-circuit voltage of 650 V. However, the output power density was found to be approximately 0.05 mW/cm^2^, which could be improved. To improve the performance of TENGs, researchers have composited MXenes with or embedded in polymers. The presence of MXenes in polymer films has two effects, dielectric permittivity and percolation, that need to be balanced by the percentage of MXenes (Figure 6) to obtain the maximum performance.^94^

PDMS is one of the most commonly used polymers to composite with MXenes.^98−101^ Liu et al. reported a PDMS/MXene (4:1) triboelectric layer for use in TENGs^99^ that can achieve outputs of 453 V and 132 μA. He and co-workers^98^ studied different compositions of PDMS/MXene where the weight concentration of the MXene was tuned between 1% and 5%. TENGs fabricated with a spin-coated PDMS/MXene triboelectric layer can achieve an output power density of up to 10 mW/cm^2^.

Fluoropolymers such as PTFE,^96^ PVDF-TrFE,^103^ and PVDF^94,106^ have also been used in composites with MXenes. Theoretical analysis was reported by Zhang et al.,^105^ who indicated that the percentage of MXenes in the PVDF film can be tuned to maximize the output. Experimentally, 10% MXene in the PVDF film provided an output of 0.22 mW/cm^2^. The same percentage of MXene has also been reported by Bhatta et al.^106^ where PVDF fibers were functionalized by10% of MXene. An output of 1.1 mW/cm^2^ was obtained on the TENG operated in contact-separation mode.

Other polymers, such PVA,^107,108^ rubber,^104^ and PDOT:PSS,^105^ have also been used in composites with MXenes for use in TENGs. An output power density of 0.11 mW/cm^2^ has been reported^109^ based on a triboelectric layer of PVA/MXene fibers with 30 vol % MXene. In another report,^108^ 4 wt % MXene revealed the best performance.

In addition to the composition with polymers, MXenes can also be composited inorganic materials for use in TENGs. Feng and co-workers^110^ reported an alternate-layered MXene composite produced with Nb_2_CT_*x*_ and Ti_3_CT_*x*_. They found that 15 wt % Nb_2_CT_*x*_ in the composite could lead to the best performance of the TENGs.

The electrical conductivity of MXenes makes them not only good triboelectric materials but also alternative electrodes in TENGs. Cao et al.^111^ produced a type of MXene liquid electrode for improving the performance of a TENG working in a single electrode mode. Enhanced outputs up to 300 V and 0.37 μA/cm^2^ were achieved.

A summary of the performances of the TENGs made of MXenes and composites is given in Table 4.

**Table 4:** Performances of MXene Constituted TENGs

MXene (or composites)	counter tribolayer	voltage (V)	current	power	ref
MXene/PTFE	Cu	397	21 μA	0.09 mW/cm^2^	(96)
MXene	PET	650	7.5 μA	0.65 mW	(97)
3D MXene/PDMS	Nylon	45	0.6 μA		(95)
MXene/PDMS	PET	80^a^			(101)
MXene/PDMS	PDMS	453	131 μA		(99)
MXene/PDMS	Skin	225	30 μA/cm^2^	10 mW/cm^2^	(98)
PVDF-TrFE/MXene	Nylon	270	140 mA/m^2^	4.02 W/m^2^	(103)
PVDF/MXene	Nylon	724	163.6 μA	11.213 W/m^2^	(100)
MXene/PVA	Kapton	230	270 nA	0.33 W/m^2^	(102)
MXene/Ecoflex	Nylon	790	183 μA	9.24 W/m^2^	(104)
MXene-PDOT:PSS	PTFE	29.56			(105)

a Data read from the figures in the paper.

#### 3.3.3 2D Semiconductors

2D semiconductors used in TENGs are mostly transition metal dichalcogenide (TMD) materials, including MoS_2_, WS_2_, MoSe_2_, and WSe_2_. The use of these 2D materials could benefit the theoretical understanding of the mechanism of triboelectrification. Seol and co-workers^83^ made a triboelectric series of 2D layer materials and pointed out that the triboelectric effects of the 2D studied triboelectric materials are obviously related to the effective work functions. According to the maximum output voltage and current, the 2D materials can be listed in the order of (−) MoS_2_, MoSe_2_, WSe_2_, and WS_2_ (+).

**Figure 7:**
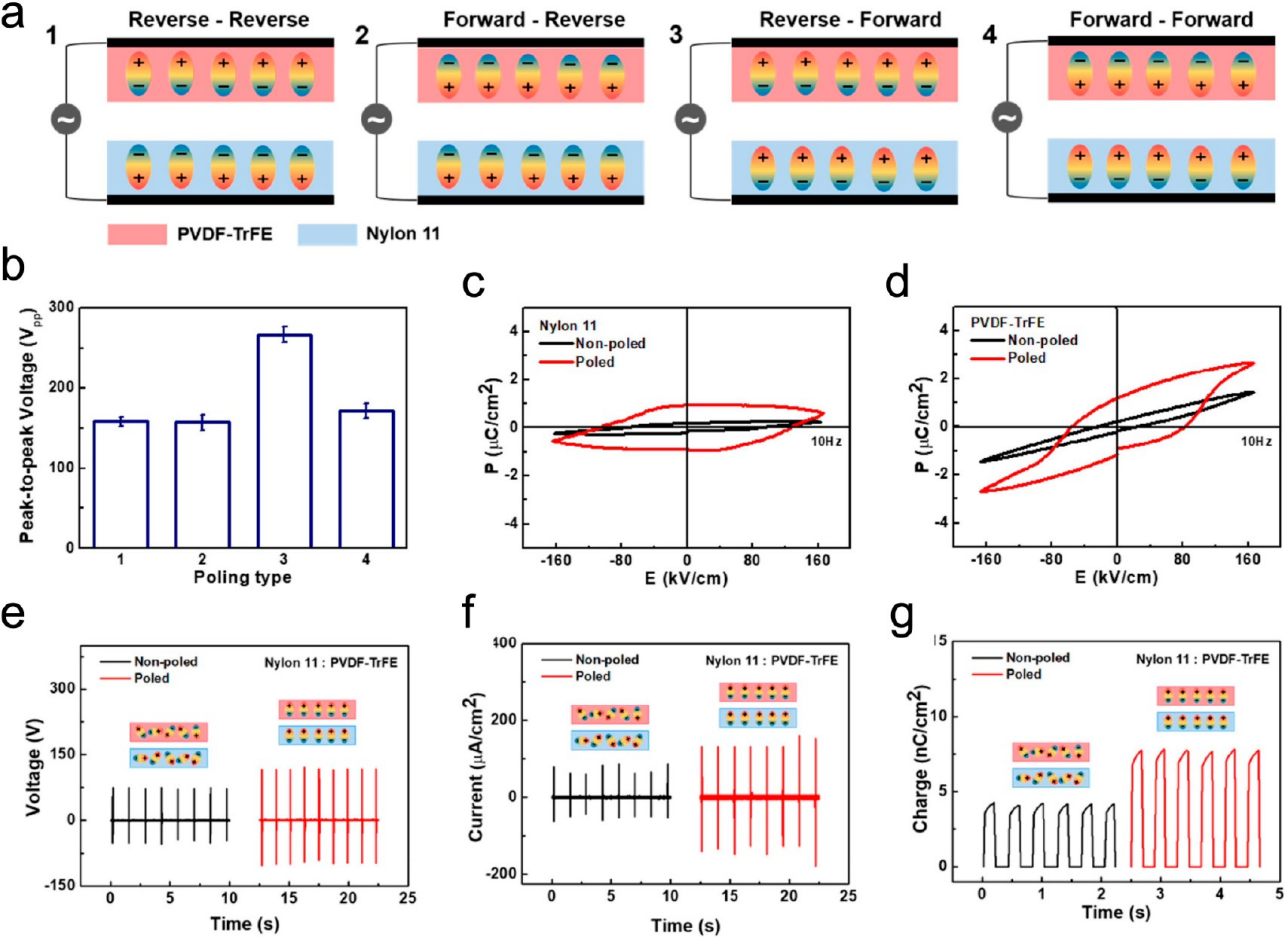
(a) Schematic drawings of four different polarization pairs. (b) Comparison of the output peak-to-peak voltages for each poling pair. (c) *P*–*E* curves of nonpoled nylon-11 and poled nylon-11. (d) *P*–*E* curves of nonpoled PVDF-TrFE and poled PVDF-TrFE. The enhancement in (e) output voltage, (f) current density, and (g) charge density before (black colored line) and after (red colored line) polarization of the friction surfaces. Reproduced from ref (102). Copyright 2019 American Chemical Society.

Among these 2D semiconductors, MoS_2_ is the most investigated in TENGs. MoS_2_ materials can act solely^112,113^ as a triboelectric layer for energy conversion or be composited with other polymers.^114−116^ The output power densities of the MoS_2_ constituted TENGs range from 74 nW/cm^2^ to 50 mW/cm^2^, while the open-circuit voltage ranges from 2.3 to 145 V. The highest output, 50 mW/cm^2^, is from a TENG^102^ with both triboelectric layers composited with MoS_2_ flakes and polarized afterward (Figure 7).

The unique semiconductive property of MoS_2_ leads to different electrical contacts in the TENGs. In some cases,^116^ the depletion layers that formed between MoS_2_ and other materials, such as Au and PPy, could enhance the output of the TENGs. This mechanism can have an impact on other 2D semiconductors but needs further study.

### 3.4 Perovskites/Ferroelectric Nanomaterials

**Figure 8:**
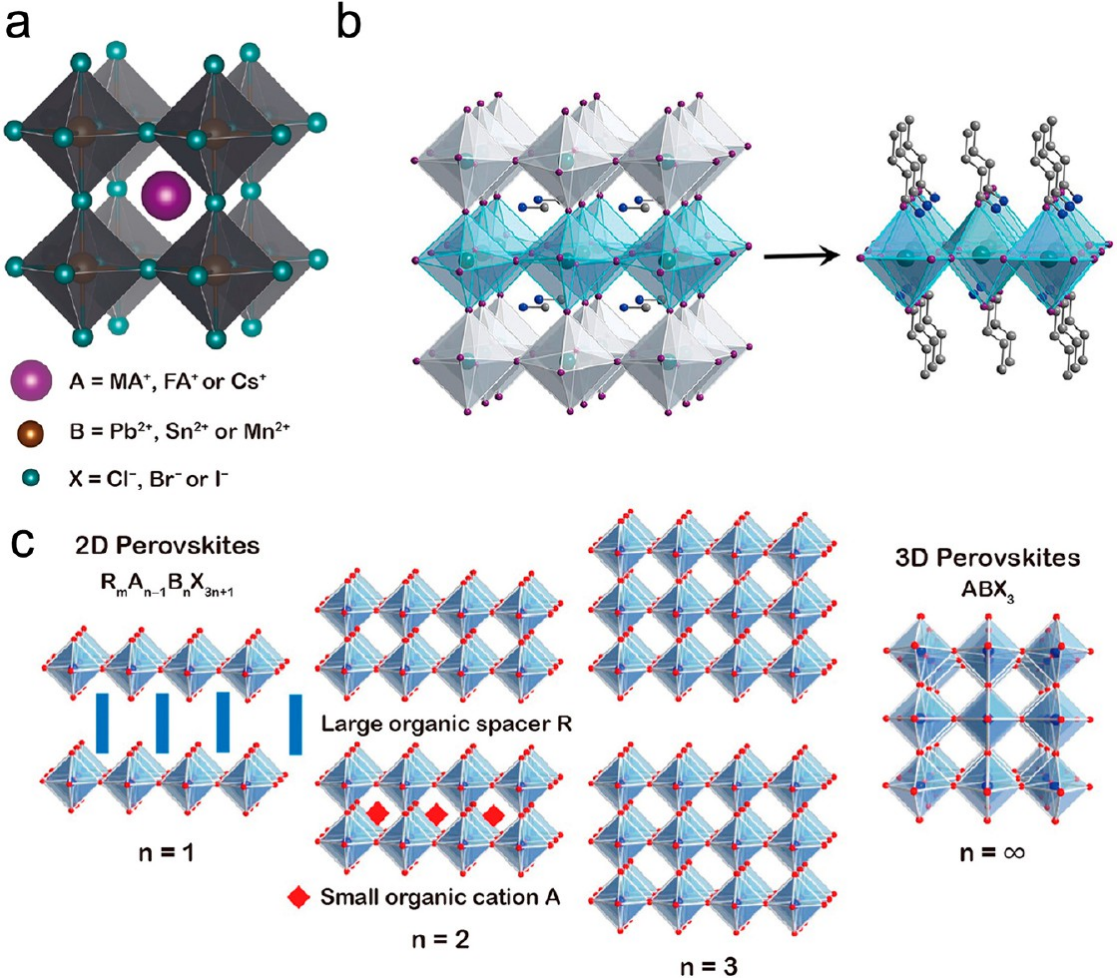
(a) Schematic representation of the typical 3D perovskite structure ABX_3_. A cation is adopted into the body center of the cubic structure formed by eight corner-sharing [BX_6_]^4–^ octahedra. (b) Schematic representation of a 3D perovskite MAPbI_3_ sliced into a 2D perovskite (C_4_H_9_NH_3_)_2_PbI_4_ along the (001) crystallographic plane. (c), Structures of the 2D perovskites of R_*m*_A_*n*–1_B_*n*_X_3*n*+1_ (from *n* = 1 to *n* = ∞) in different numbers of inorganic metal-halide layers. Reference (117), reused under CC BY 4.0.

Perovskites (Figure 8) have recently gained much attention due to their successful applications^117^ in energy harvesting technologies, including solar cells and TENGs. Recently, Ippili et al.,^118^ have reviewed the progress of the halide perovskite-based triboelectric self-powered sensors, showing the great potential of the materials. In most of the studies, single crystalline system of perovskites have been utilized, while the binary crystalline system of perovskites remains are less focused.

Many perovskites are also ferroelectric. In some papers, perovskites are emphasized, while others emphasize ferroelectricity. Therefore, we created a combined section for perovskites and ferroelectric nanomaterials.

Originally, perovskite refers to calcium titanate (CaTiO_3_). Recently, it refers to the class of compounds that have the same type of crystal structure, ^XII^A^2+VI^B^4+^X^2–^_3_. The “A” in the form can be either metals or organic groups. However, more perovskite structures have been synthesized recently that have different formulas, where “B” can combine two or more elements, such as Sr_3_Co_2_WO_9_.^119^

#### 3.4.1 Lead Halide Perovskites

The organolead halide perovskite MAPbI_3_ was first used in a triboelectric photodetector in 2015.^120^ Taking advantage of the excellent light absorption capability^121^ of MAPbI_3_, enhanced performance has been achieved on a TENG, where the open-circuit voltage, short-circuit current, and amount of electric charge have been increased by 11%, 11%, and 9%, respectively. The output increase was further improved by using a hybrid perovskite structure,^122^ MAPbI_*x*_Cl_3–*x*_. The structure couples the triboelectric and photoelectric conversion mechanisms and realizes enhancements of 55.7%, 50.8%, and 58.2%. Although the advantages of organolead halide perovskites in TENGs have been demonstrated in these studies, the output, especially the power density, remains modest.

The performance of TENGs has recently been improved by using inorganic lead halide perovskites, such as CsPbBr_3_,^124^ CsPbBr_2.6_I_0.4_,^125^ Co(OH)(CO_3_)_0.5_/Pt/CsPbIBr_2_,^126^ and Ba^2+^-doped CsPbBr_3_.^127^ It seems that CsPbBr_3_ has the best performance in TENGs with or without doping compared to the other perovskites. In the case of doping, the electron binding energy, surface potential, and dielectric property of CsPbBr_3_ can be optimized to obtain the best performance. For example, a TENG^127^ made of CsPb_0.91_Ba_0.09_Br_3_ resulting from Ba^2+^ doping showed a power density of 3.07 W/m^2^, short current density of 22.8 mA/m^2^, and open-circuit voltage of 200 V.

A recent study has shown that the halogen elements in perovskites can be tuned to change their polarizabilities. The polarizability of CsPbBr_3_ is calculated at 0.47 using density function theory calculations, while it is 0.52 for CsPbCl_3_.^128^ Spontaneous polarization of the perovskites could further enhance their built-in electric field, which could lately improve the triboelectrostatic electric field by retaining more triboelectric surface charges.

#### 3.4.2 Lead-Free Perovskite/Ferroelectric Materials

In many reports, lead-free perovskites are referred to as ferroelectric materials instead. Therefore, we use ferroelectric herewith.

Ferroelectric materials such as BaTiO_3_ exhibit spontaneous electric polarization that can be reversed by an external electric field. Spontaneous polarization gives the material an excellent pyroelectric effect that can be used in energy harvesting for different applications.^129,130^

**Figure 9:**
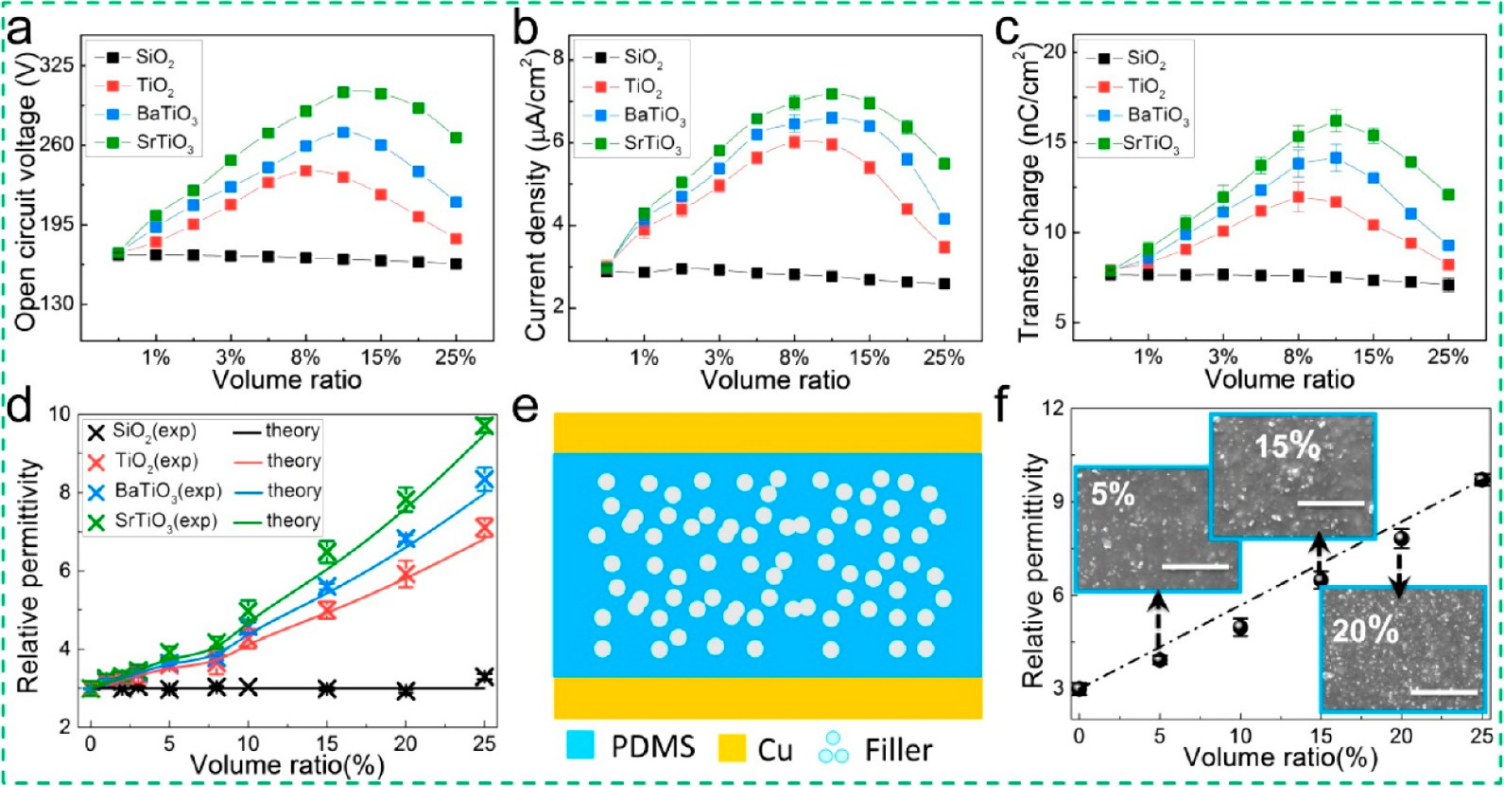
Electrical measurements of each as-fabricated film-based TENG (*f* = 2.5 Hz). (a) Open-circuit voltage, (b) short-current density, and (c) transfer charge of the SiO_2_/TiO_2_/BaTiO_3_/SrTiO_3_-filled samples with various volume ratios. (d) Comparison of the measured results with effective medium theoretical calculations. (e) Schematic diagram of the composite film. (f) Relative permittivity changes as a function of SrTiO_3_ content from 0 to 25 vol %. The insets show SEM images of composite films at various volume ratios. The scale bars are 1 μm. Reproduced from ref (123). Copyright 2016 American Chemical Society.

The application of ferroelectric materials in TENGs has recently gained increasing interest from researchers. In most studies, ferroelectric materials are composited with a polymer to form a triboelectric layer. The first application of such a layer in TENGs was reported in 2015,^123^ where BaTiO_3_ (<100 nm) and SrTiO_3_ (<100 nm) nanoparticles were filled into sponge PDMS films (Figure 9). The high permittivity and the nanopores created by the nanoparticles resulted in enhanced performance of the TENG (SrTiO_3_ filled) with a charge density of ∼19 nC/cm^2^, a maximum open-circuit voltage of 338 V, and a maximum power density of 0.647 mW/cm^2^. The BaTiO_3_-filled PMDS film yielded a charge density of ∼14.7 nC/cm^2^ at a volume ratio of 10%. In another report^131^ in which 120 nm sized BaTiO_3_ nanoparticles were used to fill a PDMS film, the optimized volume ratio was 30%. An output power density of 0.14 mW/cm^2^ was obtained on the TENG. For composites with PVDF,^132^ the optimized ratio of BaTiO_3_ was 11.25%, which led to an open-circuit voltage of 131 V and a short-circuit current density of 89 μC/m^2^. The performance was further boosted to 161 V and 112 μC/m^2^ by reducing the composite layer thickness down to 5 μm. A more complex composite created by core–shell structured BaTiO_3_–poly(*tert*-butyl acrylate) nanoparticles and PVDF^133^ has also been studied as a triboelectric material. The composite has a higher dielectric constant of 26.5 at 150 MV/m, which contributes to the high output of the TENG, which is 2.5 times higher than that of the pure PVDF-based TENG.

In addition to engineering polymers, BaTiO_3_ has also been composited with natural polymers such as cellulose for use in TENGs. Through simple vacuum filtration, BaTiO_3_ particles can be tracked among the cellulose fibers forming a composite film.^134^ The presence of the BaTiO_3_ particles increased the dielectric constant of the cellulose film, which improved the performance of the TENG assembled with the composite film. The optimized ratio of BaTiO_3_ particles was 13.5%, which yielded an open-circuit voltage of 181 V, short-circuit current of 21 μA, and power density of 4.8 W/m^2^. The open-circuit voltage and the short-circuit current were enhanced by 150% and 310%, respectively. The addition of silver nanowires to cellulose–BaTiO_3_ composites^135^ could increase the conductivity of the composite film and could be used as both a triboelectric layer and an electrode. Simple poling treatment of the composite can further promote the TENG performance. An output power density of 180 μW/cm^2^ was achieved with the film paired with fluorinated ethylene propylene (FEP). Moreover, long-term stability was realized on the TENG with no output reduction after 10 000 cycles. A similar enhancement of poling has also been found in a BaTiO_3_/PDMS-composited triboelectric film.^131^ Recently, a type of cellulose/BaTiO_3_ aerogel paper^136^ was made that uses BaTiO_3_ to increase the permittivity and charge trapping capability.

The composition of BaTiO_3_ nanoparticles and inorganic nanomaterials has also been studied to enhance the output of TENGs. Taking advantage of the synergistic effect of multiwalled carbon nanotubes and 70 nm sized BaTiO_3_ nanoparticles,^137^ the charge and power density of a TENG was boosted to 160 μC/m^2^ and 204 μW/cm^2^. Larger sized BaTiO_3_ particles (500 nm) lead to lower output, which indicates that the larger surface area of the nanoparticles plays an important role in the output enhancement. The reason for the size effect is that the smaller nanoparticles create a larger interfacial volume fraction between BaTiO_3_ and PDMS.

**Figure 10:**
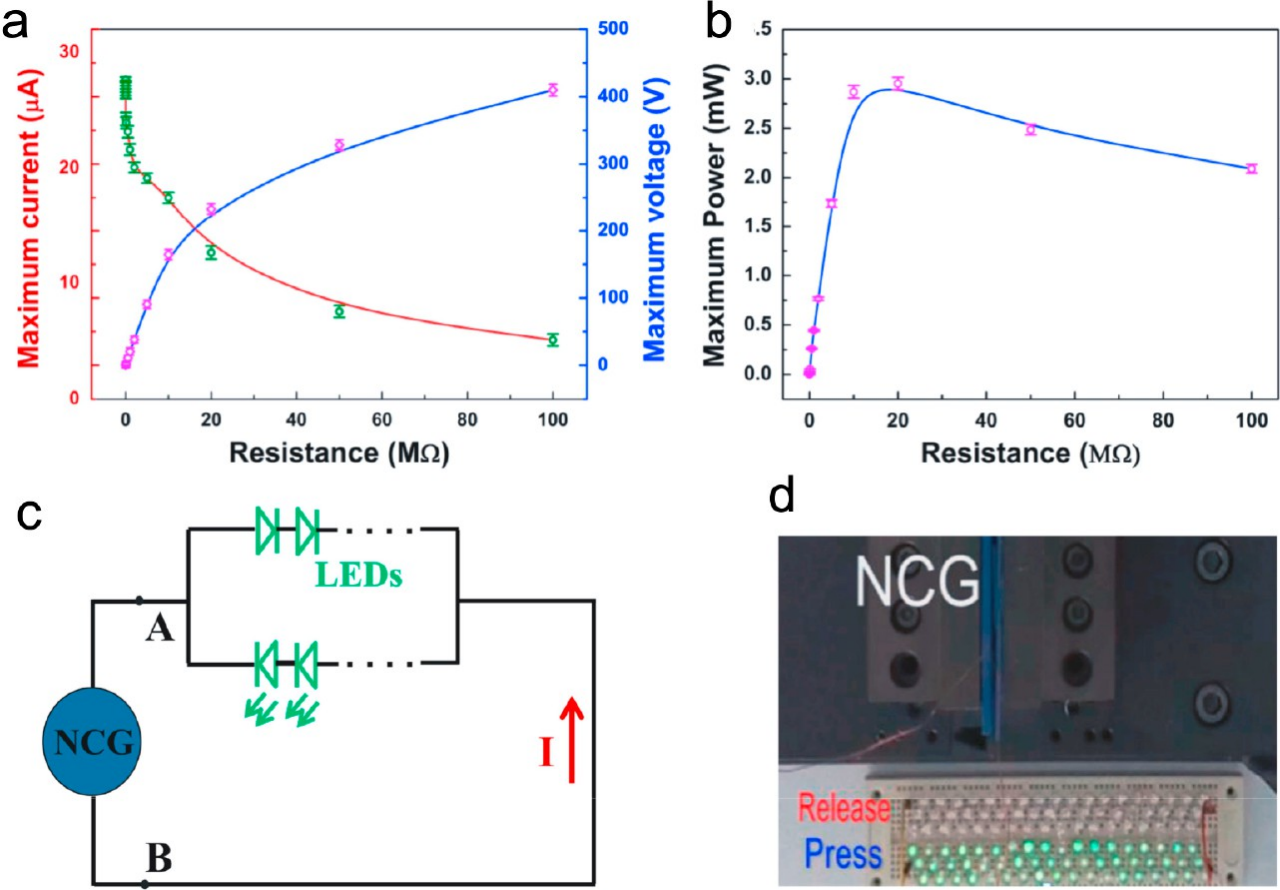
ZnSnO_3_ nanocube–PDMS-composited TENG. (a) Measured load voltage and current under outer variable resistance from 10 Ω to 600 MΩ. (b) Relationship between the instantaneous power outputs and load resistance. The effective power was harvested up to 3 mW at a load resistance of ∼20 MΩ. Circuit diagram (c) and photograph (d) of blue LEDs being lit that are powered directly by the TENG. Reproduced from ref (140). Copyright 2015 Elsevier.

Researchers have also studied other ferroelectric materials, such as BiFeO_3_ and ZnSnO_3_. A film made of a BiFeO_3_-modified glass fiber fabric on a PDMS^138^ layer has been studied as a triboelectric material for use in wearable hybrid nanogenerators. The nanogenerator can generate an open-circuit voltage of 110 V and a short-current density of 3.67 μA/cm^2^ at a low frequency of 1 Hz. The addition of BiFeO_3_ increased the dielectric constant from approximately 2.75 to 4, which led to an output power density of 151.42 μW/cm^2^. Such a power output is based not only on the triboelectricity of the layer but also on the piezoelectricity. A similar approach has also been applied for ZnSnO_3_ constituted TENGs,^139^ where a piezoelectric membrane of PVDF–ZnSnO_3_ and PA6 has been used as a triboelectric layer. The composite shows a piezoelectric coefficient *d*_33_ of −65 pm/V, which is significantly higher than that of the pure PVDF film. With the enhancement from the piezoelectric effect, the TENG made of the composite film generated a maximum open-circuit voltage of 520 V and a short-circuit current density of 2.7 mA/m^2^. Another report (Figure 10) that uses smaller ZnSnO_3_ nanocubes^140^ showed a higher short-circuit density of 7 μA/cm^2^ and output power of 3 mW. Such an output can easily drive 106 blue LEDs. Mechanisms of the higher output have been suggested from the designed macrostructures and controlled microdielectric materials of the TENG.

Table 5 shows the summary of the performances of the perovskite- or ferroelectric nanomaterial constituted TENGs.

**Table 5:** Performances of Perovskite and Ferroelectric Nanomaterial Constituted TENGs

perovskite/ferroelectric nanomaterials	counter tribolayer	voltage (V)	current	power	ref
MAPbI_3_	PTFE	22.8	0.92 μA	12.5 μW	(122)
CsPbBr_3_	PVDF	240	4.13 μA/cm^2^	3.31 W/m^2^	(124)
CsPbBr_2.6_I_0.4_	PVDF	192	16.7 μA	1.2 W/m^2^	(125)
Co(OH)(CO3)0.5/Pt/CsPbIBr2	PVDF	243	3.1 μA/cm^2^	2.04 W/m^2^	(126)
Ba^2+^ doped CsPbBr_3_	PVDF	220	22.8 mA/m^2^	3.07 W/m^2^	(127)
CsPbCl_3_	PVDF	258	30 μA^a^	3.06 W/m^2^	(128)
BaTiO_3_/PDMS	Al	375	6 μA	2.25 mW	(131)
BaTiO_3_/PVDF	Nylon	161	6.2 μA	225.6 mW/m^2^	(132)
BaTiO_3_–P*t*BA	Al	35^a^	2.1 μA/cm^2^	224 mW/m^2^	(133)
BC/BaTiO_3_	PDMS	181	21 μA	4.8 W/m^2^	(134)
BaTiO_3_/BC/Ag NWs	PTFE	460	23 μA	180 μW/cm^2^	(135)
Cellulose/BaTiO_3_	PDMS	88	8.3 μA	141 μW	(136)
PVDF–ZnSnO_3_	PA-6	520	2.7 mA/m^2^	0.47 mW/m^2^	(139)
ZnSnO_3_–PDMS	Al	400	7 μA/cm^2^	3 mW	(140)

a Data read from the figures in the paper.

### 3.5 Carbon Nanomaterials

Carbon nanomaterials exist in different crystal structures and different dimensions and have different electric and dielectric properties. Therefore, the application of carbon nanomaterials in TENGs can vary based on the properties that have been utilized. Carbon nanomaterials can be amorphous or crystalline depending on their chemical structures. Carbon black is one of the typical amorphous carbons. Crystalline carbon includes graphene, graphite, carbon nanotubes, fullerenes, and diamonds.

Graphene and graphene oxide as 2D nanomaterials have been reviewed above and are excluded in this section. However, graphene quantum dots as 0D nanomaterials are included in this section. Graphite, as an important member of the carbon material family, is also reviewed here for its application in TENGs, although it is generally not considered a nanomaterial.

#### 3.5.1 Amorphous Carbon

Amorphous carbon is usually produced by using flame methods that result in powders containing nanosized carbon particles. High-temperature carbonization has also been used for producing amorphous carbon.^141^ Amorphous carbon can be directly deposited on a p-type Si wafer for use as a triboelectric layer^142^ that has resulted in an open-circuit voltage of 8.5 V, a short-circuit current density of 0.24 μA/cm^2^, a power density of 0.5 mW/cm^2^, and an instantaneous energy conversion efficiency of 7.71%. The output was further enhanced by embedding graphene sheets in the amorphous carbon layer. The embedded graphene sheets contribute two effects: an edge effect that can capture electrons and a channel effect that enhances the electron mobility in the layer. Such contribution has enhanced the output to 13.5 V, 0.35 μA/cm^2^, 0.63 mW/cm^2^, and 8.61%.

Carbon black is characterized under an electron microscope as nanosized particles. The materials can contain more than 97% amorphous carbon that has a high surface-area-to-volume ratio as well as good electric conductivity. Carbon black is usually composited with another material to form a triboelectric film. The presence of carbon black can change the permittivity^143^ and the work function^61^ of the composited film. When composited with PDMS, the charge storage characteristics of a TENG can be improved because the composite can store dynamically inserted negative charges. Such an improvement can further lead to a long-term increase in electrical charges. The content of carbon black in the composite has been experimentally found to be important since it plays a role in trapping charges. The carbon black content of 1 wt % resulted in the best performance of the TENG: 235 V, 35.6 μA/cm^2^, and 0.133 mW/cm^2^. Physically mixed carbon black, ZnO nanorods, and PTFE have been used to coat nickel foams for use in TENGs.^61^ Carbon black, in this case, can affect the work function of ZnO nanorods because conductive electrons can transfer from carbon black to ZnO. The optimized content of carbon black here was 20 wt %, which led to an open-circuit voltage of 28 V, a short-circuit current density of 4.5 μA/cm^2^, and a power density of 80 μW/cm^2^.

#### 3.5.2 Graphene Quantum Dots

Graphene quantum dots (GQDs) are nanosized layered carbon structures that have many activated parts due to dangling bonds at the edge. Such dangling bonds allow the GQDs to bind to organic and inorganic structures.

The addition of GQDs to a silver nanowire network can significantly enhance electron transfer.^144^ The mechanism of the enhancement is that the electron flows to the GQDs and then to the PDMS. Such an intermediation by the GQDs reduces the barrier caused by the band mismatch of the silver and the PDMS. The enhancement of the GQDs increased the sensitivity of the triboelectric electronic skin 3 times.

GQDs can be doped and embedded in PVDF to make composite nanofibers using an electrospun technique.^145^ Nitrogen-doped GQDs are negatively charged and can form ion–dipole interactions with the positive −CH_2_ dipoles in the PVDF chains. A content of 5% GQDs in the composite fibers produces the highest output of 2.7 μW/cm^2^.

#### 3.5.3 Carbon Nanotubes

Carbon nanotubes (CNTs) are 1D carbon nanomaterials that include single-walled, double-walled, and multiwalled carbon nanotubes.

**Figure 11:**
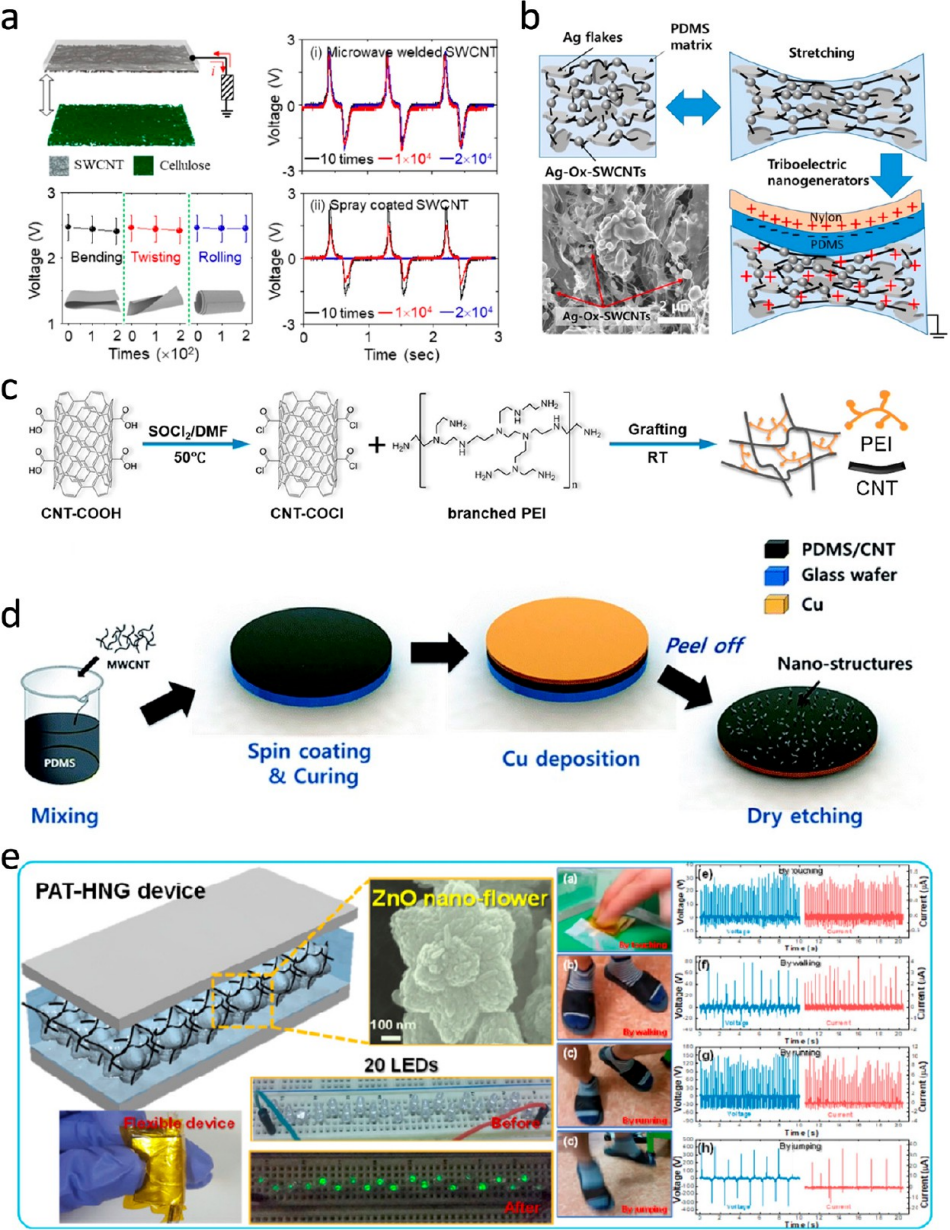
CNT constituted TENGs. (a) TENG with a welded SWCNT electrode. Reproduced from ref (146). Copyright 2019 Elsevier. (b) Stretchable TENG. Reproduced from ref (147). Copyright 2021 Elsevier, (c) Cross-link of CNT and PEI. Reproduced from ref (148). Copyright 2021 American Chemical Society, (d) Compositing of CNTs with PDMS. Reference (149), reused under CC BY 3.0. (e) PAT-HNG. Reproduced from ref (67). Copyright 2018 American Chemical Society.

A single-walled CNT (SWCNT) network layer has been used as a p-type semiconductor to form a metal–semiconductor junction^150^ with an aluminum electrode. Such a junction can reduce the loss of triboelectric charge because the SWCNT layer acts as a hole transporting layer that affects the triboelectric charge separation. The presence of the SWCNT layer can improve the charge-repelling force and the hole-blocking barrier at the interface. A wearable TENG was made based on the structure that achieved an output voltage of ∼760 V, a current of ∼51 μA, and a power density of 0.77 mW/cm^2^. The SWCNT network layer has also been used directly as an electrode.^151^ However, the loss of contact among the nanowires may cause the loss of conductivity during the operation. A microwave-assisted welding process can fix SWCNTs on a polycarbonate film (Figure 11a) and can significantly enhance the stability of the electrode.^146^ The stability allows the electrode to be used on flexible substrates for harvesting energy. For using SWCNT-based electrodes in cases where stretchability is required, a simple compositing process has been used to create a stretchable electrode together with silver flakes and PDMS (Figure 11b).^147^ A stretchable TENG working in the freestanding triboelectric layer mode was made with the electrode showing a peak power density of 84.4 mW/cm^2^.

Multiwalled CNTs (MWCNTs) have been more popularly used than SWCNTs because they have electrical properties similar to those of SWCNTs but at a lower cost. MWCNTs have been used either as a form of pure layer or as a type of composite. The deposition of a MWCNT layer can enhance the contact area of the triboelectric interface.^152,153^ Such an effect, plus the positive surface of the MWCNTs, can enhance triboelectric charge generation. The synergistic effects enhanced the output by a factor of 7-fold. By screen printing a layer of MWCNTs on a thermoplastic polyurethane nanofiber membrane (TPUNM), a type of stretchable TENG^154^ has been made where the MWCNTs act as both an electrode material and a triboelectric layer. A maximum voltage of 218 V, a current density of 0.75 μA/cm^2^, and a power density of 22.5 mW/cm^2^ were obtained on the TENG by dip coating CNTs on a fiber with a silver nanowire network layer.

Using the surface chemistry of MWCNTs, cross-linked structures of MWCNTs and other materials, such as PEI (Figure 11c), have been fabricated.^148^ The introduction of amide groups from PEI increases the tribopositivity, which further enhances charge transfer during the triboelectrification process. The results of the enhancements are represented by a 10-fold improvement in the output voltage and current.

Instead of chemical treatment of the MWCNTs for functionalization, direct mixing of the MWCNTs with polymers is less specific but more convenient experimentally. A MWCNT/PDMS composite can be simply produced by mixing the MWCNTs in PDMS kits (Figure 11d).^149,155,156^ The addition of nanoflower-like ZnO (Figure 11e)^67^ to the composite film can take advantage of the piezoelectric properties of ZnO to boost the output of a hybrid nanogenerator, namely, PAT-HNG. By mixing MWCNTs with PVA and PDAP, which resulted in dual responsive hydrogels,^39^ self-healable and deformable TENGs have been fabricated that can survive under 200% strain. The mixing of ureidopyrimidinone-functionalized MWCNTs with IU-PAM could also produce self-healable TENGs.^38^ By compositing with PANI, a PANI-MWCNT film-based TENG^157^ could sense ammonia gas with a detection limit of 0.01 ppm.

#### 3.5.4 Graphite

Graphite is generally not considered a nanomaterial, although nanosized graphite materials have been widely used. Here, we review the use of graphite in TENGs because the material has great importance in several aspects, such as a nonmetal electrode material, an environmentally friendly material, a low-cost material, and an oxidation resist material.

**Figure 12:**
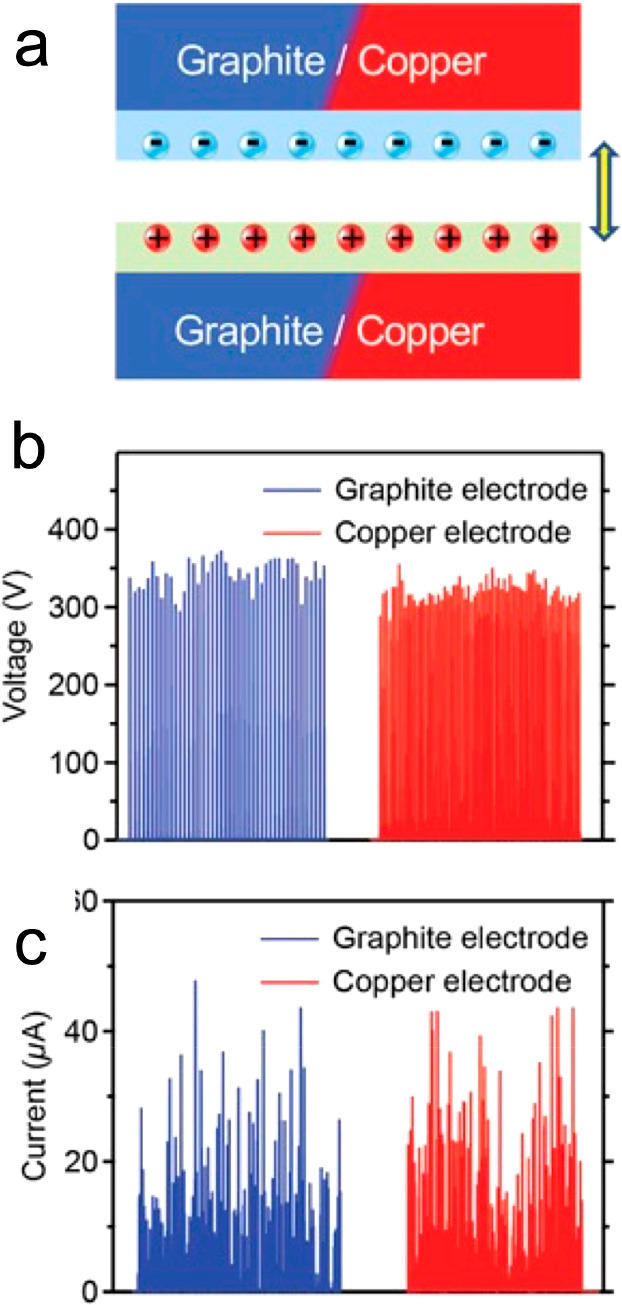
(a) Schematic drawing of an evaluation that compares the graphite electrodes used in the FG-TENG and copper electrodes. (b) Voltage measured on the two TENGs using graphite or copper electrodes. (c) Current measured on the two TENGs using graphite or copper electrodes. Reference (158), adapted and reused under CC BY 4.0.

In most cases, graphite is used as the electrode material. As an electrode material,^159^ a thick graphite film has excellent conductivity and mechanical strength that in practice could be directly used as an electrode that can allow the TENG to have the same or even better performance than metallic electrodes. A fully green TENG (FG-TENG)^158^ using only graphite as the electrode material has been reported recently. The output power of the FG-TENG using the graphite electrodes was 35% higher than that using the copper electrode (Figure 12). A thin graphite layer could also be coated on other substrates, such as sandpaper,^160^ pristine paper,^161^ and paper cards,^162^ with coating methods of brushing, rod coating, and drawing, respectively.

In addition to its role as an electrode, graphite has also been composited with PDMS to form triboelectric layers for use in TENGs.^163,164^ A graphite to PDMS ratio of 2:5 could result in the highest output of the TENG,^150^ where the maximum open-circuit voltage is 410 V and the short-circuit current is 42 μA.

#### 3.5.5 Other Carbon Nanomaterials

In addition to the above reviewed carbon materials, other carbon nanomaterials, such as fullerene^165^ and diamond-like carbon,^166,167^ have also been applied in TENG studies. The high electron affinity of the fullerene^165^ could enhance the output of the TENG to a maximum open-circuit voltage of ≈1.6 kV, a short-circuit current of ≈100 μA, and a power density of 38 W/cm^2^. A TENG made of a diamond-like carbon film^153^ had a maximum open-circuit voltage of 38 V, a short-circuit current of 3.5 μA, and a power density of 57 mW/cm^2^.

There are also studies on 3D-structured carbon nanomaterial-based TENGs that enrich the application of carbon nanomaterials. A rubber/carbon nanofiber-composited 3D structure^168^ was produced and applied as a TENG operating in single electrode mode with an open-circuit voltage of 91 V and a short-circuit current of 2.87 μA. By using the templating method, patterned 3D carbon electrodes^169^ have been made with the aim of increasing the contact area with the counter triboelectric layers. Compared to metallic electrodes, 3D carbon electrodes have shown more robustness to humidity and mechanical frictions.

## 4 Conclusions and Perspectives

The applications of TENGs include energy harvesting, a variety of sensor types, biomedical applications, the Internet of Things (IoT), and human–computer interactions. Different applications have different requirements for the materials and production technologies.^170,171^ For example, a wearable TENG requires flexible materials. Many of the requirements could not be fulfilled by macroscale materials but by nanosized materials. Therefore, different types of nanomaterials have been studied to determine their applications in TENGs. The utilization of inorganic nanomaterials in TENGs has been proven very successful. The benefits of inorganic nanomaterials include the following:

(1) Increase the contact area of the triboelectric surfaces. The high surface area to volume ratio significantly increases the contact area.
(2) Tune the dielectric properties of the composited triboelectric materials. Inorganic nanomaterials include many types of materials with dielectric properties, such as dielectric constants, spread in a very large range. By selecting the type, size, and content of the inorganic nanomaterials, the dielectric properties of the triboelectric materials could be tuned as expected.
(3) Enhance the charge transportation. The interface between a triboelectric material and an electrode may have a barrier due to the misalignment of the band structure. The presence of the nanomaterials could reduce the barrier so that the charges could be transported more conveniently.
(4) Tune the optical properties of the TENGs. Networks of nanowires such as silver and CNT nanowires can be used to fabricate transparent electrodes for use in TENGs.
(5) Mechanical properties: flexibility and stretchability. The network structures of the nanowires enabled the triboelectric films to be flexible and stretchable but retained their electrical properties.
(6) Hybrid nanogenerators. Some inorganic nanomaterials, such as ferroelectric nanomaterials, have piezoelectric properties that allow the fabrication of hybrid nanogenerators.

Although many works reviewed here have explored the advances of inorganic nanomaterials in TENGs, there are still some issues that need to be addressed in the future:

(1) Quantitative understanding of the relationship between the performances of the TENGs and the sizes of the inorganic nanomaterials. Theoretical models need to be developed and optimized in the future.
(2) Theoretical models of how the size and percentage of inorganic nanomaterials change the dielectric properties of the composites.
(3) Applications of new types of inorganic nanomaterials^172^ in TENGs. Only a small portion of inorganic nanomaterials have been studied in the last several years, requiring more effort.

In summary, we have reviewed the recent advances of inorganic nanomaterials in triboelectric nanogenerators based on the roles, types, and characteristics of nanomaterials. The advantages of inorganic nanomaterials and the performance of TENGs promoted by inorganic nanomaterials have been reviewed. Some prospective studies have been suggested that could inspire future research in the area. This Review provides an overview of how and why inorganic nanomaterials are utilized in TENGs, which offers guidance for future studies.

## Abbreviations

TENG: triboelectric nanogenerator
NW: nanowire
CVD: chemical vapor deposition
PDMS: polydimethylsiloxane
PEDOT:PSS: poly(3,4-ethylenedioxythiophene) polystyrenesulfonate
PTFE: polytetrafluoroethylene
PVDF: polyvinylidene fluoride
PVA: poly(vinyl alcohol)
PDAP: photothermally active polydopamine particles
IU-PAM: imine bond and UPy unit, −polyazomethine
ITO: indium tin oxide
SEM: scanning electron microscope
TEM: transmission electron microscope
KPFM: Kelvin probe force microscope
CNT: carbon nanotube
SWCNT: single-walled carbon nanotube
MWCNT: multiwalled carbon nanotube
GO: graphene oxide
rGO: reduced graphene oxide
IoT: Internet of things
GQD: graphene quantum dots
PA-6: polyamide 6
PtBA: poly(*tert*-butyl acrylate)
BC: bacterial cellulose
